# Pharmacokinetics and safety comparison of cycloserine capsules and their generic counterpart: a randomized, open-label, two-period, two-sequence, single-dose crossover bioequivalence study in healthy Chinese subjects

**DOI:** 10.3389/fphar.2026.1801953

**Published:** 2026-08-31

**Authors:** Xiuqing Peng, Liying Zhang, Caiyun Jia, Congyang Ding, Haojing Song, Zhanjun Dong, Huizhen Wu, Wanjun Bai

**Affiliations:** Phase I Clinical Trial Laboratory; Hebei Key Laboratory of Clinical Pharmacy, Hebei General Hospital, Shijiazhuang, China

**Keywords:** bioequivalence, cycloserine, pharmacokinetics, safety, tuberculosis

## Abstract

**Introduction:**

Cycloserine is a broad‐spectrum antibiotic and clinically important second‐line anti‐tuberculosis agent. This study assessed the bioequivalence of a test cycloserine formulation versus its reference formulation under fasted and fed conditions.

**Methods:**

Using a randomized, open‐label, two‐period, two‐sequence crossover design, 64 healthy Chinese participants were enrolled (63 completed). Subjects received a single 250‐mg oral dose of both formulations. Bioequivalence was evaluated using plasma concentration‐time profiles and key pharmacokinetic (PK) parameters. Statistical analysis employed the Average Bioequivalence (ABE) method. Geometric mean ratios (GMRs) and 90% confidence intervals (90% CIs) for C_max_, AUC_0‐t_ and AUC_0‐∞_ fell entirely within the predefined range (80.00–125.00%) under both conditions.

**Results:**

Both formulations exhibited comparable safety profiles.

**Discussion:**

Bioequivalence was established between the test and reference cycloserine capsules under fasted and fed states.

**Clinical Trial Registration:**

Chinese Clinical Trial Registry (www.chinadrugtrials.org.cn) as (CTR20233940).

## Highlights

Analyses were performed using.

- Liquid chromatography-tandem mass spectrometry (LC-MS/MS) system (SCIEX).
- Refrigerated high-speed centrifuge (Model: Multifuge X Pro Series, Thermo Scientific).
- Analytical balance (Model: XPE26, Mettler Toledo).
- Multi-tube vortex mixer (Model: HD-2500, Hangzhou Youning Instrument).

## 1 Introduction

Tuberculosis (TB), which is primarily caused by *Mycobacterium tuberculosis*, remains a significant global health burden. In China, TB accounts for approximately 25,000 annual deaths, exceeding the combined mortality rate from other infectious diseases (Trajman et al., 2025; Guo et al., 2024; Long et al., 2021; Freschi et al., 2021). Cycloserine, a second-line anti-tuberculosis drug, exerts antibacterial effects by inhibiting the synthesis of the mycobacterial cell wall. Its unique targets (D-alanine racemase and D-alanyl-D-alanine synthetase) confer activity even against drug-resistant strains, making it particularly suitable for treating multidrug-resistant tuberculosis (MDR-TB) following the failure of first-line drugs (such as isoniazid and rifampicin). Cycloserine is designated as a core drug for treating drug-resistant TB in World Health Organization (WHO) (2019) and Chinese (2019) guidelines (Deshpande et al., 2018; Alghamdi et al., 2019). Globally, approximately 500,000 drug-resistant TB cases emerged in 2024, with China reporting a 7.1% resistance rate (Farhat et al., 2024; Dheda et al., 2024; Vasiliu et al., 2024).

The reference product (Meiji Seika Pharma, Japan) is unavailable in China, necessitating a reliance on generic alternatives. Per China National Medical Products Administration (NMPA) requirements, generic cycloserine must demonstrate bioequivalence (BE) to the innovator product prior to market approval to ensure therapeutic equivalence. Furthermore, BE studies can also investigate the pharmacokinetics and safety profile of cycloserine in the Chinese population (Sun et al., 2022). This study evaluated the BE and safety of a domestic cycloserine capsule (Hebei Longhai Pharmaceutical Co., Ltd.) against the reference formulation in healthy Chinese subjects under fasted and fed conditions, following NMPA guidelines.

## 2 Methods

### 2.1 Study drugs and reagents

This study utilized two cycloserine capsule formulations:

Test formulation: Cycloserine capsules (250 mg per tablet, batch number: 23090174, assay: 99.4%), were manufactured by Hebei Longhai Pharmaceutical Co., Ltd. Reference formulation: Cycloserine capsules (250 mg per tablet, batch number: 00,505, assay: 98.9%), were manufactured by Meiji Seika Pharma Co., Ltd., Japan.

The following analytical standards were employed:

Chemical reference standard: Cycloserine (batch number: SGLC23-889R, purity: 98.0%, source: Shimadzu (Shanghai) Global Laboratory Consumables Co., Ltd).

Internal standard: Cycloserine-15 N,d3 (batch number: 4-SNM-44–3, chemical purity: 98%, isotopic purity: 98.9%, source: Toronto Research Chemicals).

### 2.2 Ethics approval and study population

The trial was conducted at the Phase I Clinical Research Center of Hebei General Hospital between December 2023 and February 2024, and registered at the Chinese Drug Clinical Trial Registration Platform (http://www.chinadrugtrials.org.cn/, Registration No. CTR20233940). The study was complied with the China NMPA’s *Good Clinical Practice* (GCP) guidelines, *Declaration of Helsinki* and *ICH Harmonised Guideline for GCP.* The protocol and amendments received approval from Hebei General Hospital’s Ethics Committee (Approval No. 2023–37, 2023–37-01).

Healthy male and female volunteers aged ≥18 years with body mass index (BMI) between 19.0 and 26.0 kg/m^2^ (≥50 kg for males and ≥45 kg for females) were eligible for the trial. All subjects underwent comprehensive assessments including physical examination, vital signs measurement, medical history review, laboratory tests (hematology, biochemistry, urinalysis), 12-lead electrocardiogram (ECG), chest X-ray urine drug screen and alcohol breath test.

Subjects with the following conditions were excluded for the study: acute/chronic diseases not suitable for the trials, abuse of alcohol, smoking (>5 cigarettes/day) or drugs, allergy to cycloserine or any ingredient in the tablet. Subjects with a history of epilepsy, depression, severe anxiety, or psychiatric disorders were also excluded. In addition, participants who had consumed any CYP450-affecting foods (e.g., grapefruit, pomelo, mango, pitaya, starfruit) within 48 h pre-dose, taken any medication within 28 days prior to the trial, participated in any other clinical trials within 3 months, or underwent blood donation or loss ≥400 mL within 3 months are refused to join in the study. Besides, subjects who were pregnant, breastbreeding or planning to become pregnant were also excluded.

As for the informed consent, written informed consents were obtained from all participants after detailed explanation of study objectives, procedures, and potential risks/benefits. Subjects retained the right to withdraw at any time without penalty.

### 2.3 Study design

This study was a single-center, randomized, open-label, two-period, single-dose, crossover bioequivalence trial. The research comprises two independent trials: one under fasted conditions and the other under fed conditions. Using SAS statistical software (v9.4), a randomization schedule was generated to assign fasted trial subjects to sequence A (Test Formulation-Reference Formulation, T-R) or B (R-T) in a 1:1 ratio. Subjects were administered either the test or reference formulation (250 mg) with 240 mL of water on Day 1 (Period 1) and Day 8 (Period 2) after fasted for at least 10 h. Similarly, fed trial subjects were randomly assigned to sequence C (T-R) or D (R-T) in a 1:1 ratio. They consumed a standardized high-calorie, high-fat meal (800–1,000 kcal: 150 kcal protein, 250 kcal carbohydrates, 500–600 kcal fat) 30 min before administration on Day 1 (Period 1) and Day 8 (Period 2), followed by the test or reference formulation (250 mg) with 240 mL of water.

In both fasted and fed trials, water intake was restricted for 1 h before and after dosing (except for the 240 mL administered with the drug). Standard lunches and dinners were provided at 4 and 10 h post-dose, respectively, with identical meals maintained across both periods. A 7-day washout period separated the two treatment periods.

### 2.4 Plasma sample collection and analysis

Fasted Trial: Blood samples (approximately 4 mL each) were collected at 19 time points per period: pre-dose (0 h, within 1 h before dosing) and at 15 min, 30 min, 45 min, 1 h, 1.25 h, 1.5 h, 1.75 h, 2 h, 2.5 h, 3 h, 4 h, 6 h, 8 h, 10 h, 12 h, 24 h, 48 h, and 72 h post-dose.

Fed Trial: Blood samples (approximately 4 mL each) were collected at 19 time points per period: pre-dose (0 h, after the high-fat meal, and before dosing) and at 30 min, 1 h, 1.5 h, 2 h, 2.5 h, 3 h, 3.5 h, 4 h, 4.5 h, 5 h, 6 h, 7 h, 8 h, 10 h, 12 h, 24 h, 48 h, and 72 h post-dose.

Samples were drawn into precooled EDTA-K_2_ anticoagulation vacuum tubes, gently inverted several times for mixing, and immediately placed upright in an ice bath before centrifuged at 2500 g ± 10g, 4 °C for 10 min in an hour. The separated plasma supernatant was aliquoted into test tubes (approximately 0.8 mL) and backup tubes. Plasma aliquots were immediately stored at −80 °C (range: 90 °C to −60 °C) within 1 h post-centrifugation until bioanalysis.

Protein precipitation method was used for pre-treatment of the plasma samples. Sample nebulization and ionization were performed using an electrospray ionization (ESI) source, and sample concentrations were determined in the multiple reaction monitoring (MRM) mode. A validated liquid chromatography-tandem mass spectrometry (LC-MS/MS) method was employed to determine cycloserine plasma concentrations. Quantitative analysis of the target compound was performed using gradient elution and MRM. Chromatographic separation was achieved using an XBridge® Amide column (2.1 × 50 mm, 3.5 μm) with gradient elution. The mobile phase consisted of phase A (aqueous solution containing 0.02% acetic acid and 10 mM ammonium acetate) and phase B (acetonitrile solution containing 0.02% acetic acid, 10 mM ammonium acetate, 3% methanol, and 2% water). The flow rate was maintained between 0.6 and 0.8 mL/min. Mass spectrometric detection was performed using a triple quadrupole mass spectrometer (API 5500). Data acquisition and analysis were conducted using Analyst software (version 1.6.3, SCIEX, U.S.A.). Linear regression and concentration calculations were performed using Watson LIMS software (version 7.5 SP1, Thermo Fisher Scientific, U.S.A.). All computational parameters (Mean, Standard Deviation (SD), Coefficient of Variation (CV), Relative Error (RE), Matrix Factor (MF), Internal Standard-normalized Matrix Factor (ISnorm-MF), and Accuracy), excluding the DEV for calibration standards and quality control samples, were calculated using Microsoft Office Excel (version 2010 or later).

### 2.5 Safety evaluation

The safety of the two formulations was evaluated by monitoring all adverse events (AEs) occurring in subjects during the clinical study. Assessments included clinical symptoms, abnormal vital signs, and abnormal findings from laboratory tests (hematology, urinalysis, clinical chemistry, and coagulation panel) and 12-lead electrocardiogram (ECG). Laboratory tests, physical examinations, and ECG were performed at the baseline visit and 72 h after the second dose administration. Vital signs (body temperature, pulse, and blood pressure) were measured with subjects in a seated position within 1 h prior to dosing and at 2.0 ± 0.5 h, 6.0 ± 0.5 h, 12.0 ± 0.5 h, 24.0 ± 0.5 h, 48.0 ± 1.0 h, and 72.0 ± 1.0 h post-dose. All AE terms were coded using the ICH Medical Dictionary for Regulatory Activities (MedDRA) (M1). The severity of AEs was graded according to the National Cancer Institute Common Terminology Criteria for Adverse Events version 5.0 (NCI CTCAE v5.0). For each AE, the following were recorded: clinical manifestations, severity, onset time, end time, management measures, outcome, and assessment of relationship to the investigational product.

### 2.6 Statistical analysis

PK Parameter Calculation was performed via Phoenix WinNonlin software (version 8.4; Pharsight Corp., Mountain View, CA, USA). Other Statistical Analyses was performed using SAS software (Windows version 9.4; SAS Institute Inc., Cary, NC, USA). Plasma Concentration-Time (c-t) Data Analysis was performed based on PK Concentration Set - PKCS), individual and mean plasma concentration-time curves were plotted, and plasma concentration statistics (mean, SD, median, maximum, minimum, and CV) at each nominal time point were reported. As for PK Parameter Analysis, pharmacokinetic parameters (such as C_max_, AUC_0-t_, ${\text{AUC}}_{0-\infty }$ , T_max_, t_1/2_) for each subject were calculated using noncompartmental analysis (NCA). Statistics (arithmetic mean, SD, CV, median, max, min, geometric mean) for each PK parameter were reported. Bioequivalence (BE) Assessment was conducted by the following methods, the primary PK parameters (C_max_, AUC_0-t_, ${\text{AUC}}_{0-\infty }$ ) were log-transformed, and multifactor analysis of variance (ANOVA) was performed for significance testing. The model included fixed effects: treatment sequence, formulation, and period; and a random effect: subject (nested within sequence). The geometric mean ratio (GMR) (Test [T]/Reference [R]) and its 90% confidence interval (CI) were calculated for each primary PK parameter. Bioequivalence was considered if the 90% CI for the GMR of C_max_, AUC_0-t_, and ${\text{AUC}}_{0-\infty }$ were entirely contained within the equivalence range of 80.00%–125.00%. The within-subject CV for the primary PK parameters was calculated.

## 3 Results

### 3.1 Subject demographic characteristics

A total of 223 subjects were screened (fasted group: n = 109; fed group: n = 114), with 32 subjects enrolled per group (fasted: female/male = 2/30; fed: female/male = 5/27). One subject in the fasted group (K029, sequence R-T) withdrew after completing Period one dosing and 12-h blood sampling. All fed-group subjects completed the study. All fasted subjects were included in Full Analysis Set (FAS), Safety Set (SS), PK Concentration Set (PKCS), PK Parameter Set (PKPS), and Bioequivalence Set (BES) (AUC data excluded for K029). All 32 fed subjects were included in all analysis sets. All subjects met inclusion criteria. Baseline characteristics are presented in Table 1.

**TABLE 1:** Demographic baseline.

Variable	Fasted trial		Fed trial	
Age (years)				
Mean ± SD	30.4 ± 6.57		30.7 ± 8.95	
Median	31.0		30.0	
Min; max	20.44		19.45	
Sex n (%)				
Male	2 (6.3)		5 (15.6)	
Female	30 (93.8)		27 (84.4)	
Ethnicity n (%)				
Ethnic han	32 (100.0)		31 (96.9)	
Others	0		1 (3.1)	
Height (cm)				
Mean ± SD	171.5 (6.18)		171.2 (8.93)	
Median	171.0		172.3	
Min; max	160, 185.5		148, 186.5	
Weight (kg)				
​	Male	Female	Male	Female
Mean (Std)	60.0 (5.23)	67.5 (6.10)	56.7 (3.35)	68.2 (6.95)
Median	60.0	66.9	54.4	67.8
Min; max	56.3, 63.7	56.8, 77.5	52.4, 60.4	55.9, 84
BMI (kg/m^2^)				
Mean (Std)	22.8 (1.68)		22.6 (1.69)	
Median	22.9		22.6	
Min; max	19.7, 25.5		20.1, 26	

**FIGURE 1:**
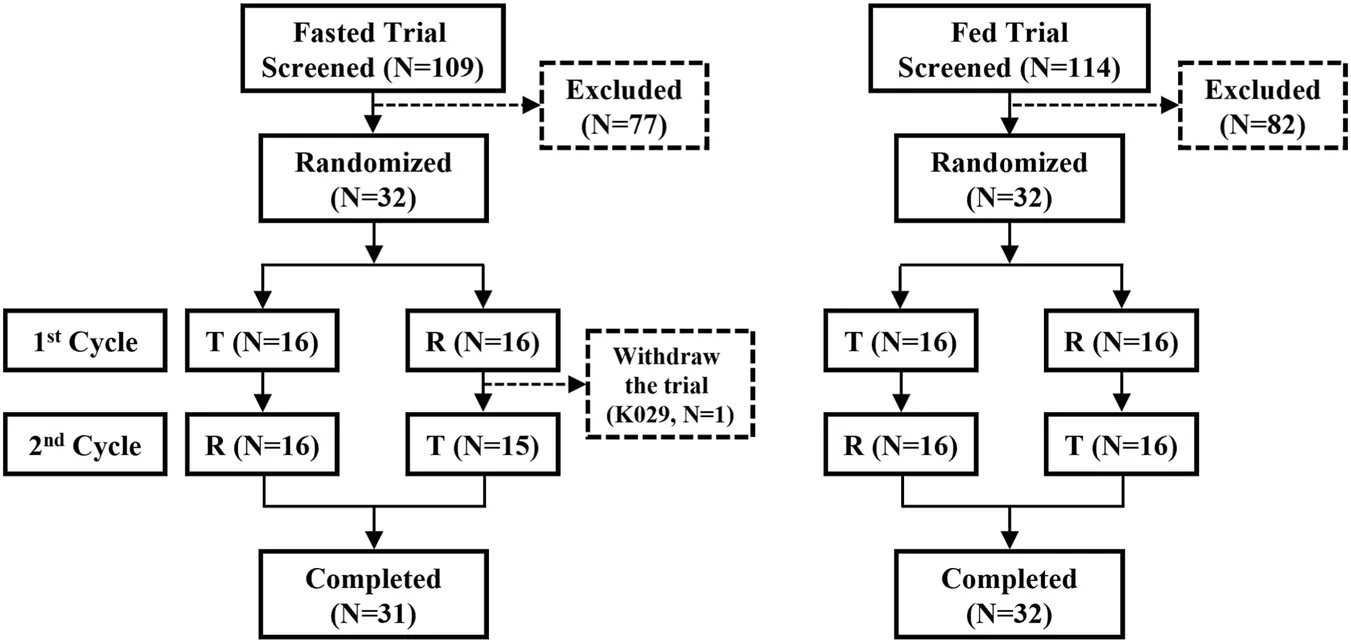
Subject flow chart. Flow chart of the trials in fasted and fed state.

Subject disposition is detailed in Figure 1.

### 3.2 Pharmacokinetic (PK) results

**FIGURE 2:**
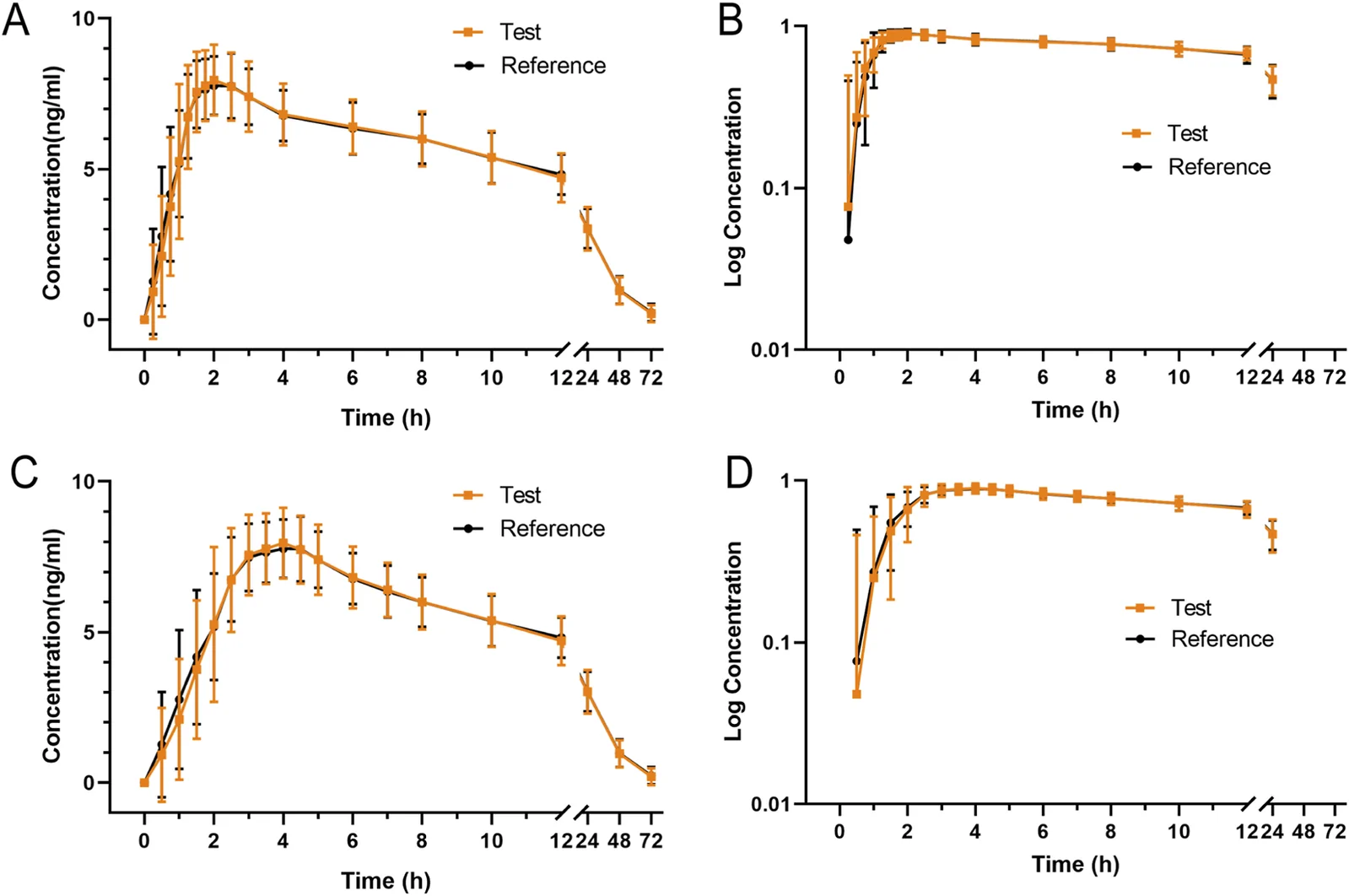
Pharmacokinetic profiles of test and reference cycloserine formulations under fasted and fed conditions. **(A)** Mean plasma concentration-time profiles (±SD) of cycloserine after single-dose administration of test and reference formulations in fasted state. **(B)** Corresponding semilogarithmic plots of data in **(A)**. **(C)** Mean plasma concentration-time profiles (±SD) of cycloserine after single-dose administration of test and reference formulations in fed state. **(D)** Corresponding semilogarithmic plots of data in **(C)**.

For the test and reference formulations in the fasted trial, the mean ± SD C_max_ values were 13.91 ± 2.58 μg/mL and 14.13 ± 2.64 μg/mL, respectively. The mean ± SD AUC_0–t_ values were 219.92 ± 43.34 h μg/mL and 208.51 ± 50.02 h μg/mL, respectively. For the test and reference formulations in the fed trial, the mean ± SD C_max_ values were 8.41 ± 1.53 μg/mL and 8.28 ± 1.08 μg/mL, respectively. The mean ± SD AUC_0–t_ values were 171.11 ± 37.13 h μg/mL and 173.76 ± 36.50 h μg/mL, respectively. The mean plasma concentration-time profiles of cycloserine under fasted and fed conditions were presented in Figure 2. Detailed PK parameters are listed in Table 2.

**TABLE 2:** Summary of pharmacokinetic parameters for cycloserine test and reference formulations under fasted and fed conditions.

Pharmacokinetic (PK) parameters	Fasted trial		Fed trial	
	Test (N = 31)	References (N = 32)	Test (N = 32)	References (N = 32)
T_max_(h)^*^	0.49 (0.49.1.24)	0.49 (0.24.1.49)	3.99 (1.49.5.99)	3.99 (0.99.5.00)
C_max_(μg/mL)	13.91 ± 2.58	14.13 ± 2.64	8.41 ± 1.53	8.28 ± 1.08
AUC_0-t_(h*ug/mL)	219.92 ± 43.34	208.51 ± 50.02	171.11 ± 37.13	173.76 ± 36.50
$\text{AU}C_{0-\infty }$ (h*ug/mL)	233.06 ± 45.69	224.06 ± 47.27	184.30 ± 39.31	186.59 ± 39.36
AUC__%Extrap_(%)	5.64 ± 2.35	7.73 ± 7.31	7.18 ± 2.70	6.84 ± 2.36
t_1/2_(h)	15.23 ± 2.52	15.204 ± 2.74	14.58 ± 2.94	14.78 ± 3.09
λ_z_(1/h)	0.05 ± 0.01	0.05 ± 0.01	0.05 ± 0.01	0.05 ± 0.01

^*^ Medium (min, max).

### 3.3 Bioequivalence (BE) results

The primary PK parameters for cycloserine test versus reference formulations (C_max_, AUC_0-t_, and ${\text{AUC}}_{0-\infty }$ ) are presented in Table 3. As the intra-subject CV for each parameter was less than 30% under both fasted and fed conditions, bioequivalence was assessed using average bioequivalence (ABE) criteria. These results demonstrate that the 90% CIs for all primary PK parameters fell within the 80.00%–125.00% bioequivalence limits under both conditions (Table 4). Besides, no statistically significant difference in T_max_ was observed between formulations by the signed-rank test (P > 0.05) (Table 5). Collectively, these findings indicate bioequivalence between the test and reference cycloserine formulations under fasted and fed states.

**TABLE 3:** Bioequivalence assessment of cycloserine test versus reference formulation under fasted and fed conditions.

Bioequivalence determination of fasted trial						
PK parameters	T	R	GMR (%)	90%CI	CV (%)	Bioequivalence
	GM(N)	GM(N)				
C_max_ (ug/mL)	13.69 (31)	13.88 (32)	98.60	92.26–105.39	15.60	Yes
AUC_0-t_ (h*ug/mL)	215.54 (31)	208.02 (31)	103.62	100.31–107.03	7.51	Yes
$\text{AU}C_{0-\infty }$ (h*ug/mL)	228.51 (31)	222.76 (31)	102.58	100.00–105.23	5.92	Yes

Bioequivalence determination of fed trial						
PK parameters	T	R	GMR (%)	90%CI	CV (%)	Bioequivalence
	GM(N)	GM(N)				
C_max_ (ug/mL)	8.29 (32)	8.21 (32)	101.01	96.65–105.58	10.44	Yes
AUC_0-t_ (h*ug/mL)	167.16 (32)	170.09 (32)	98.28	94.75–101.94	8.64	Yes
${\text{AUC}}_{0-\infty }$ (h*ug/mL)	180.17 (32)	182.63 (32)	98.65	95.15–102.27	8.51	Yes

**TABLE 4:** Analysis of variance (ANOVA) for log-transformed pharmacokinetic parameters of cycloserine in fasted and fed bioequivalence studies.

Effect factor	Fasted trial			Fed trial		
	Ln (C_max_)	Ln (AUC_0-t_)	$\mathrm{Ln}\left({\text{AUC}}_{0-\infty }\right)$	Ln (C_max_)	Ln (AUC_0-t_)	Ln ( ${\text{AUC}}_{0-\infty }$ )
Sequence	0.0982	0.1790	0.1705	0.2064	0.8986	0.8067
Formulation	0.6588	0.0725	0.1006	0.7010	0.4270	0.5270
Period	0.4243	0.1136	0.0857	0.0525	0.4279	0.5012

**TABLE 5:** Adverse events (AEs) of cycloserine test versus reference formulation under fasted and fed conditions.

Parameter	Fasted group (n = 32)	Fed group (n = 32)
Subjects with AEs	10 (31.3%)	16 (50.0%)
Total AE incidents	11 events	27 events
AE severity*	All grade 1 (mild)	24 events: Grade 1 (Mild) 3 events: Grade 2 (moderate)
AEs requiring intervention	0 events	0 events
Drug-related AE events	1 event	2 events**
Adverse reaction rate	3.1% (1/32 subjects)	3.1% (1/32 subjects***)

* Graded per CTCAE v5.0. Drug-relatedness determined by investigators.

** Both drug-related AEs, occurred in the same subject.

*** Refers to subject count, not event count.

To assess the impact of extrinsic factors (period, formulation, and sequence) on bioequivalence evaluation, we performed a multivariate analysis of variance (ANOVA) using a linear mixed-effects model (fixed effects: period, formulation, sequence; random effect: subject) on natural log-transformed primary pharmacokinetic parameters for each state separately. Results demonstrated no statistically significant differences for sequence, formulation or period on the outcomes in either the fasted or fed state (P > 0.05 for all factors in both states) (Table 4).

### 3.4 Safety results

Both formulations demonstrated acceptable safety profiles under fasted and fed conditions, with no new safety concerns identified. Adverse event (AE) incidence rates were comparable between conditions (31.3% vs. 53.1%; P = 0.08), and severity profiles were consistent (Table 5; Table 6). In the fasted state, all AEs resolved without medical intervention. In the fed state, moderate AEs (Grade 2) resolved without intervention. No serious AEs occurred.

**TABLE 6:** Summary of AEs.

Parameter	Fasted trial						Fed trial					
	T (N = 31)			R (N = 32)			T (N = 32)			R (N = 32)		
	n	%	E	n	%	E	n	%	E	n	%	E
Sum	6	19.4	6	4	12.5	5	9	28.1	14	8	25.0	13
AE severity												
Grade 1	6	19.4	6	4	12.5	5	9	28.1	14	6	18.8	10
≥Grade 2	0	0	0	0	0	0	0	0	0	2	6.3	3
Correlation with drugs												
Possible related	1	3.2	1	0	0	0	1	3.1	2	0	0	0
Possible unrelated	5	16.1	5	4	12.5	5	8	25.0	12	8	25.0	13
Adverse event outcomes												
Urine leukocytes positive	0	0	0	0	0	0	1	3.1	2	1	3.1	2
Neutrophil count decreased	0	0	0	0	0	0	1	3.1	1	0	0	0
Blood triglycerides increased	2	6.5	2	4	12.5	4	2	6.3	2	4	12.5	4
Alanine aminotransferase increased	1	3.2	1	0	0	0	1	3.1	1	0	0	0
Blood fibrinogen decreased	1	3.2	1	0	0	0	1	3.1	1	0	0	0
Blood creatinine increased	1	3.2	1	0	0	0	2	6.3	2	0	0	0
00Aspartate aminotransferase increased	0	0	0	0	0	0	1	3.1	1	0	0	0
Blood creatine phosphokinase increased	0	0	0	1	3.1	1	0	0	0	0	0	0
Blood bilirubin increased	0	0	0	0	0	0	0	0	0	1	3.1	1
Conjugated bilirubin increased	0	0	0	0	0	0	0	0	0	1	3.1	1
Blood glucose increased	0	0	0	0	0	0	1	3.1	2	0	0	0
Blood potassium decreased	1	3.2	1	0	0	0	0	0	0	0	0	0
Upper respiratory tract infection	0	0	0	0	0	0	1	3.1	1	0	0	0
Dental abscess	0	0	0	0	0	0	0	0	0	1	3.1	0
Adverse event outcomes												
Recovered/Resolved	2	6.5	2	3	9.4	4	5	15.5	6	5	15.5	6
Ongoing	0	0	0	0	0	0	0	0	0	0	0	0
Improved/Resolving	1	3.2	1	1	3.1	1	0	0	0	1	3.1	2
Worsened	0	0	0	0	0	0	0	0	0	0	0	0
Unknown/Lost to follow-up	3	9.7	3	0	0	0	6	18.8	8	2	6.3	5

n = number of subjects reporting adverse event(s), E = number of adverse event incidents reported.

## 4 Discussion

Cycloserine has been established by the World Health Organization (WHO) as a core second-line drug for the treatment of multidrug-resistant tuberculosis (MDR-TB), playing a pivotal role in both long-course (18–20 months) and short-course (6–9 months) regimens. This designation is based on its unique antibacterial mechanism, specifically the competitive inhibition of key cell wall synthesis enzymes (D-alanine synthetase and L-alanine racemase), which blocks cell wall synthesis in *M. tuberculosis* and other pathogens, and its low propensity for inducing resistance. Robust clinical efficacy is evidenced by significantly higher treatment response rates (89.4% vs. 70.2%, P < 0.05), accelerated sputum culture conversion (68.1% at 3 months, 80.9% at 6 months), and superior radiographic outcomes (lesion absorption, cavity closure) compared to controls in MDR-TB patients (Li et al., 2019; Chirehwa et al., 2020; Yu et al., 2018; Gao et al., 2021).

Despite the Chinese anti-TB drug market exceeding ¥200 million (approximately $28 million USD) in 2025, access to cycloserine remains constrained by the high cost and non-importation of the originator product. Compounding this issue is the paucity of PK and clinical data specific to the Chinese population. Consequently, the development and rigorous bioequivalence (BE) assessment of domestic generic cycloserine formulations represent a significant clinical imperative. Successfully establishing bioequivalence not only facilitates affordable access but also forms the essential foundation for systematically characterizing cycloserine’s PK profile in Chinese patients. This knowledge is vital for optimizing dosing strategies, enhancing medication safety, accelerating domestic drug development, and ultimately advancing global TB control objectives in alignment with the WHO’s “End TB Strategy” (Zhu et al., 2023; Ranjan et al., 2019; Nahid et al., 2016; Shao et al., 2011; Wu et al., 2025; Mao et al., 2025; He et al., 2024; Caño-Muñiz et al., 2018).

The present study comprehensively evaluated the bioequivalence and safety of a domestic cycloserine capsule formulation in healthy Chinese volunteers. Critically, under both fasted and fed conditions, the 90% confidence intervals (CIs) for the geometric mean ratios (GMRs) of all primary PK parameters (C_max_, AUC_0-t_, and ${\text{AUC}}_{0-\infty }$ ) were strictly contained within the stringent regulatory acceptance range of 80.00%–125.00%, unequivocally meeting the bioequivalence criteria mandated by both the NMPA and FDA. This robust finding corroborates the preliminary conclusion of Lan Huiyu et al. (Huiyu, 2018), while decisively addressing key limitations of that prior work. By employing a substantially larger sample size (n = 32 per group vs. n = 24) and a deliberate gender-balanced design (in contrast to the exclusion of females), our study provides a more reliable and generalizable evidence base to support the clinical substitution of the domestic generic product across the broader Chinese population.

The observed time to peak concentration (T_max_) of 3–4 h aligns well with established literature reporting high oral bioavailability and is consistent specifically with the fed data generated here. The study further reinforces cycloserine’s known pharmacokinetic behavior, notably its extensive tissue distribution facilitated by excellent penetration of physiological barriers, including the blood-brain barrier. The results in concentrations in cerebrospinal fluid (CSF), placenta, and breast milk are comparable to plasma levels, underpinning its utility in central nervous system TB but necessitating caution in breastfeeding women (hence their exclusion from this trial cohort). Furthermore, confirmation that cycloserine is predominantly excreted unchanged renally (∼65%) validates its applicability in urinary TB, while highlighting the critical need for dose adjustment in renal impairment. The essential role of therapeutic drug monitoring (TDM) is re-emphasized by the drug’s narrow therapeutic index and the heightened risk of neurotoxicity (e.g., dizziness, depression) at serum concentrations exceeding 30 μg/mL (Deshpande et al., 2018; Zhu et al., 2024).

Methodologically, the utilization of a highly sensitive LC-MS/MS assay (LLOQ = 0.05 μg/mL) ensured precise quantification of cycloserine concentrations, significantly bolstering the reliability of the derived PK parameters and BE conclusions. Nevertheless, several pivotal research gaps demand focused attention. Firstly, our present study employed a single-dose design. While this single-dose BE study confirms fundamental equivalence, MDR-TB treatment necessitates prolonged administration (6–24 months). The potential for altered PK (e.g., accumulation, enzyme induction/saturation) or PD responses during chronic dosing remains unexplored. Comprehensive multiple-dose PK/PD studies are therefore imperative to establish the generic’s equivalence and safety profile under real-world treatment durations. Besides, the neurotoxicity thresholds is undefined. Although TDM based on total serum concentration is standard practice, the specific relationship between trough concentrations (C_min_) and the incidence/severity of neurotoxicities remains inadequately characterized. Developing a validated toxicity threshold analysis model, potentially leveraging the sensitive LC-MS/MS methodology employed here, is crucial for personalized risk mitigation and dose optimization. In addition, significant clinical populations, notably patients with hepatic or renal impairment, are systematically excluded from standard BE trials. Consequently, evidence-based dose adjustment strategies for these groups within the Chinese population are virtually non-existent. Dedicated PK studies in these vulnerable cohorts are urgently required to ensure safe and effective therapy.

Addressing these knowledge gaps will be paramount for maximizing the clinical impact of domestic cycloserine generics, ensuring optimal patient outcomes across diverse populations and treatment scenarios, and fully realizing their potential contribution to TB control efforts.

## 5 Conclusion

This study demonstrated that the domestic cycloserine capsule formulation achieved bioequivalence with the reference formulation under both fasted and fed conditions. The 90% confidence intervals for the geometric mean ratios of the primary pharmacokinetic parameters strictly complied with regulatory requirements. Both formulations exhibited favorable safety and tolerability profiles in healthy volunteers. These outcomes not only provide critical evidence to support subsequent clinical trials of the domestic cycloserine capsule but will also significantly accelerate its clinical deployment in the Chinese market, thereby addressing the accessibility gap for the non-imported originator product. By facilitating the availability of high-quality generic alternatives, this research directly enhances drug accessibility for MDR-TB treatment in China, ultimately contributing to the objectives of the WHO’s global “End TB Strategy”.

## Data Availability

The original contributions presented in the study are included in the article/supplementary material, further inquiries can be directed to the corresponding author.
